# To Mr. Potts

**Published:** 1801-11

**Authors:** T. Sheldrake

**Affiliations:** No. 50, Strand


To Mr. POTTS.

Sir,

WHEN I examined your leg in the preſence of Mr. Lynn, I did not, from motives of delicacy, attempt to aſcertain the principles principles upon which it was conſtructed; I confined myſelf to obſerve the manner in which you uſed it, and what actions you could perform with it. In conſequence of that examination, I declared my opinion that it was the moſt perfect, with reſpect to the power of imitating the natural actions of a leg in actual uſe, that I had yet ſeen; and now that I do know the principles on which it is conſtructed, I repeat my opinion with this addition, that with reſpect to the action of its joints, on which its utility chiefly depends, it is leſs likely to go out of order than any other that has been made public; in ſaying this, I make *no exception*, as I know the principles upon which every thing of this kind is conſtructed, that has for many years been offered to public notice.

In the Medical Journal I perceive that you have fallen into an error, by ſaying "it is known, that where amputation has been performed above the knee, any artificial leg which has yet been invented to ſupply the loſs, is very imperfect, as the perſon uſing it, is obliged to make a ſemi-circular motion with it in walking, &c."

This is not true. Some years ago, a Mr. Mann, who you have not perhaps heard of, invented a knee joint which certainly is not liable to that inconvenience, and at the ſame time is totally different from yours; ſeveral years before the date of his invention, things poſſeſſing ſimilar properties were made by a perſon ſtill alive here, and I know of others of ſtill older date, ſome of them as long ago as the time of my grandfather, and all of them as different from his, as his is from yours. The truth is, that when men direct their ingenuity to ſubjects on which they have not previouſly collected information, they are prone to believe that what has not been done *to their knowledge, has not been done at all*; this ſeems to have been your caſe, and I adviſe you to correct the miſtake the firſt opportunity, or you will afford ſome one an opportunity to accuſe you publicly of ſaying what is not true.

Let me adviſe you at the ſame time to avoid engaging in any public diſpute on the ſubject; your invention has merit enough to bear a compariſon with any other that is intended for the ſame purpoſes; but that compariſon ſhould be made by others, not by yourſelf: the malignity of any one who might fear that your diſcovery would diminiſh the value of his, might induce him to engage you in a diſpute from *which you could gain no advantage*, though he might hope it would be ſerviceable to him; and the impudence, the vulgarity, perhaps the want of principle in ſome of thoſe who may become your opponents, might induce them not only to load you with abuſe, but to

Mmm 2 extend extend it to gentlemen* who recommend you for no other reafon, but becaufe they think you deferve it.

You are at liberty to print this if you think it will ferve you; the facts it relates are within my own knowledge: but if you fhould ufe it as an inftrument of controverfy with any perfon whatever, I will take no notice of any remarks that may be made on it. I am, &c.

*No.* 50, *Strand, March* 5, 1801. T. SHELDRAKE.

* This feems to have been prophetic, for Mr. Lynn has fhewn me a letter in which he is treated with much fcurrility and abufe, becaufe he has recommended my invention to notice, and not that of the writer; among other curious obfervations he fays, his leg is fo perfect, that *a man may dance a hornpipe gracefully with it.* I difclaim all competition or comparifon with any perfon whatever; but if I did not, I fhould certainly acknowledge myfelf vanquifhed by one who can make a wooden leg that may be *danced with gracefully.* I. P.

